# Intestinal helminthic infections among elementary students of Babile town, eastern Ethiopia

**DOI:** 10.11604/pamj.2015.20.50.5251

**Published:** 2015-01-20

**Authors:** Ephrem Tefera, Jemal Mohammed, Habtamu Mitiku

**Affiliations:** 1 Department of Medical Laboratory Sciences, College of Health and Medical Sciences, Haramaya University, Harar, Ethiopia

**Keywords:** Intestinal helminths, soil transmitted helminths, prevalence, students

## Abstract

**Introduction:**

Intestinal helminthic infections are important public health problems in developing countries. In Ethiopia, intestinal parasitic infections are highly prevalent because of low living standards and poor environmental sanitation. There are several areas in Ethiopia from which epidemiological information is lacking including Babile town. The aim of this study was to determine the prevalence of intestinal helminthic infection among students of Babile town.

**Methods:**

A cross sectional study was conducted from May 14 to June 08, 2012. Stool samples collected from 644 students were examined by the McMaster method. Data were analyzed using SPSS version 16.0. Univariate analysis was carried out using the Chi-square test to check for presence or absence of association between exposure and the presence of infection and odds ratios with 95% CI were computed to measure the strength of association. Logistic regression was used to calculate predictors of helminthic infection. Statistical significance was set at P < 0.05.

**Results:**

The prevalence of intestinal helminths was 13.8%, of which three students were infected with soil transmitted helminths with a prevalence rate of 0.47%. The prevalence of *Hymenolepis nana, Enterobius vermicularis*, hookworm, and *Trichiura trichiura* infections were 13, 0.6, 0.3, and 0.2% respectively. Intestinal helminthic infection was significantly associated with grade and sex of the school children.

**Conclusion:**

The prevalence of intestinal helminths was low. Health information dissemination is recommended. Since infection by *Hymenolepis nana* is a long term health problem in the area, provision of regular treatment by anthelminthic drug of choice for hymenolepiasis is also recommended

## Introduction

Intestinal helminthic infections are important public health problems in developing countries [1]. They are the most common infections among school age children and they tend to occur in high intensity in this age group [2]. The World Health Organization estimates that over 270 million pre-school children and over 600 million of school children are living in areas where the parasites are intensively transmitted and are in need of treatment and preventive interventions [3]. These infections are most prevalent in tropical and subtropical regions of the developing world where adequate water and sanitation facilities are lacking [4].

The global prevalence and number of cases of intestinal helminths infection in school age children have been estimated to be Roundworm 35% (320 millions); Whipworm 25% (233 millions); Hookworm 26% (239 millions), others 14% (128millions) [5]. Other species of intestinal helminths are not widely prevalent. Intestinal helminths rarely cause death. Instead, the burden of disease is related to less mortality than to the chronic and insidious effects on health and nutritional status of the host [6, 7]. In addition to their health effects, intestinal helminth infections also impair physical and mental growth of children, thwart educational achievement, and hinder economic development [8, 9]. The high prevalence of these infections is closely correlated with poverty, poor environmental hygiene and impoverished health services [10]. Intestinal helminth infections occur in all regions of Africa; particularly in sub-Saharan Africa, they are common and of major health concerns because of factors that predispose man to the infections such as poverty, poor sanitation, ignorance and malnutrition prevail [11].

In Ethiopia, intestinal parasitic infections are highly prevalent because of low living standards, poor environmental sanitation, unsafe human waste disposal systems, inadequacy and lack of safe water supply, and low socio-economic status [12]. Report from a literature survey indicated that Ethiopia has one of the lowest quality drinking water supply and latrine coverage in the world [13]. This could be one reason for why intestinal parasitism has been widespread in Ethiopia. Moreover, parasitic helminthic infections are the second most predominant causes of outpatient morbidity in the country [12].

In Ethiopia the prevalence of *Ascaris lumbricoides* infection was 29% in the highlands, 35% in the temperate areas and 38% in the lowlands. The prevalence of hookworm infection was highest in the lowlands (24%) followed by the temperate (15%) and highland (7%); whereas *Trichuris trichiura* infection exhibited similar prevalence's in all altitudinal regions (13% on the average) [14].

So far several studies identifying the prevalence of intestinal parasites in general and the prevalence of intestinal helminths in particular have been carried out in Ethiopia [15–19]. Conducting a survey on the prevalence of various intestinal helminthic infections in different geographic regions is a prerequisite for developing appropriate control strategies. Hence, the World Health Organization (WHO) presented a simple methodology to assess the prevalence of helminths, stratified by ecozones, for settings where information is scarce [20].

Data from a study in Nigeria indicated that intestinal helminthiasis differs by sex of school children [21]. In contrast, a similar study in Gondar has shown; intestinal helminthiasis did not differ by sex of school children [17]. Previous study in Babile town reported an overall prevalence of intestinal helminthiasis to be 27.2% and the predominant species identified being *Hymenolepis nana* with a prevalence rate of 10.1% [22].

In spite of the fact that a number of studies have been undertaken over the years on the prevalence of intestinal helminths among school children in different part of Ethiopia, yet there are several areas in all the regional states from which epidemiological information is lacking including our study area, Babile town. Therefore, this study is aimed at determining the prevalence of intestinal helminthic infections among elementary students of Babile town. It is believed that this study will provide recent information for those who are working in the prevention and control of intestinal helminthiasis.

## Methods

This study was conducted in Babile town, Eastern Hararghe from May 14 to June 8, 2012. The town is located about 561 Km away from the capital, Addis Ababa. It is situated in eastern Hararghe Zone between two nearby towns named: Harar and Jigjiga. The total population of the town are 17, 704 of which 8782 are males and 8922 are females. Babile town is situated at an altitude of 1340 meters above sea level with mean annual rainfall and mean annual temperature of 410 – 800mm and 24°C – 28°C respectively. In the town there are two elementary schools (Babile Elementary School #1 & Babile Elementary School #2) and one secondary school. In the town there are 3742 students of which 2086 are males and 1656 are females. The heath coverage of the town reached almost 100%. The water and latrine coverage of the town were 28% and 90% respectively [23].

Six hundred forty four students were selected from the schools to participate in the study and the sample size was determined using the standard formula for single population proportion & the proportion of STHs among elementary students of Babile town was 14.2% (p = 0.142) [22] to get maximum sample size, expected margin of error set at 4% (d = 0.04) and with 95% confidence level. Hence, the calculated sample size becomes 644. Then, the sample size was allocated to the two elementary schools proportional to size and the sampling frame was the students’ enrollment list from the two schools. Hence according to probability proportional to size calculation Elementary school No 1 contributed 567 students of the 644 sample size and Elementary school No 2 contributed 77 of the 644 required sample size. Then the study subjects were selected from the list at random using random number table i.e. 567 students from the enrollment list of Elementary school No 1 and 77 students from the enrollment list of Elementary school No 2. All the selected 644 students were voluntary to participate in the study and hence received an interview after providing the stool sample. This makes the response rate to be 100%.

All the necessary information was collected using pre-structured questionnaire. Two Technicians from the local health center were recruited and assigned as data collectors. School teachers and unit leaders helped the data collectors in the entire data collection process.

All the time before beginning stool sample collection, the latrines were inspected for cleanliness by the data collectors to get them cleaned. After giving adequate instruction on how to collect the stool sample, each study subject was provided a stool cup and applicator stick to bring at least 3gm of fresh stool sample of his/her own, that was sufficient for egg count by the McMaster method to determine the prevalence and infection intensity of soil transmitted helminths and the prevalence of other helminthic parasites. Unit leaders of the two schools attended students who are small enough for proper stool sample collection. A laboratory Technologist supervised the appropriateness of the stool sample delivered by the study participants before accepting as a sample. After providing the required stool sample, all the students were interviewed by the data collectors for the completion of data collection. Finally each sample was labeled and transported (after being preserved using formalin) to Harar Campus side lab together with filled questionnaire for processing and examination.

All collected stool samples were processed by the McMaster Method for egg count to determine the prevalence and infection intensity of soil transmitted helminths and to determine the prevalence of other intestinal helminths [24].

The generated data were entered, cleaned and analyzed using SPSS version 16.0. Intestinal helminth prevalence was expressed as the percentage of subjects found positive for each helminth. Univariate analysis was carried out using the Chi-square test to check for presence or absence of association between each exposure and the presence of infection and odds ratios with 95% CI were computed to measure the strength of association. Logistic regression was used to calculate predictors of helminthic infection. Statistical significance was set at P < 0.05.

Ethical clearance was obtained from Institutional Research Ethics Review Committee (IRERC), College of Health and Medical Sciences, Harar campus, Haramaya University. Informed written consent from the parents of each student and verbal ascent from the students were obtained before data collection. The objective and benefit of the study was thoroughly explained to the Parents/Guardians/Teachers of the study subjects. Only volunteers were involved and students were having the right to withdraw from the study. Information meeting was held with parents/guardians/school teachers and with the students to explain the purpose of the study, and the procedure involved. Results were kept confidential. All students positive for *Hymenolepis nana* were treated with Praziquantel at a dose of 25mg/kg and those students positive for *Trichuris trichiura, Enterobius vermicularis* and hookworms were treated with Mebendazole at a dose of 100mg for three days.

## Results

### Socio-demographic characteristics

A total of 644 students from the two elementary schools of Babile town were participated in this study. Of the 644 stool specimens examined 89 were positive for one or more intestinal helminths making the prevalence rate 13.8%. Out of the 644 study participants 364 (56.5%) were males and 280 (43.5%) were females. The age range of the students varies between 5 and 25. Of which age range 5 – 14 years consists of the highest number of students 593 (92.1%) the remaining 51 (7.9%) students were found in the age range 15 – 25 (Table 1). Mean age of the students was 10.45 years + 2.9 SD. The weight of the students varies between 15kg and 70 kg with mean weight of 31.84 kg + 10.15SD. Similarly the height of the students varies between 1.04m and 1.80m with mean height of 1.38m + 14.43SD.

**Table 1:** Socio-demographic characteristics of students in Babile town, Eastern Ethiopia, 2012

Variables		Frequencies (n = 644)	%
Sex	Male	364	56.5
	Female	280	43.5
Age group	5-14	593	92.1
	15-25	51	7.9
Ethnicity	Oromo	352	54.7
	Somali	165	25.6
	Amhara	101	15.7
	Gurage	24	3.7
	Harari	1	0.2
	Tigray	1	0.2
Religion	Muslim	515	80.0
	Orthodox	99	15.4
	Protestant	30	4.7

### Prevalence of intestinal helminths

The prevalence of intestinal helminths was 13.8% (89 out of 644). The most prevalent detected parasite was *Hymenolepis nana* 13% (84/644) followed by *Enterobius vermicularis* 0.6% (4/644) and the by Hookworms 0.3% (2/644). The least prevalent was *Trichuris trichiura* 0.2% (1/644). Furthermore, the prevalence of soil transmitted helminths was 0.47% (3/644). Both hookworm infected students were with light infection intensity (100 eggs per gram of feces and 200 eggs per gram of feces respectively). Similarly *Trichuris trichiura* infected student was with light infection intensity (300 eggs per gram of feces).

### Risk factor analysis for intestinal helminthic infections

Out of the 89 positive students for intestinal helminths 41 were males and the remaining 48 were females. There is a statistical significant intestinal helminth infection difference between males and females (OR 0.61 and 95% CI is 0.39 – 0.96) and (P = 0.033) (Table 2). Out of the 89 intestinal helminthiasis infected students, 85 were found in the age range 5-14, four were in the age range 15-25. Intestinal helminthiasis infection is independent of age range of students (cruds OR 1.97 and 95% CI is 0.69 – 5.60) (P = 0.205) (Table 2). Of 89 infected students, only 2 (2.2%) students were infected with two parasites i.e. by *Enterobius vermicularis and Hymenolepis nana*. The remaining 87 (97.8%) were infected with one parasite only. Out of the 89 intestinal helmiths infected students, 26 (16.7%), 18 (17.5%), 15 (17.2%), 19 (19.6%), 3 (5.1%), 4 (7.5%), 2 (5.3%), and 2 (3.9%) students were from grades 1, 2, 3, 4, 5, 6, 7, and 8 respectively. A statistically significant intestinal helminthic infection difference was observed among the different grades (P = 0.018) Six hundred forty four students from the two elementary schools participated in this study. Of which 443 (68.8%) were first cycle students (Grades 1 - 4) and the remaining 201 (31.2%) were second cycle students (Grades 5 - 8). Out of the 89 intestinal helmiths infected students, 78 (17.6%) were first cycle students and the rest 11 (5.5%) were second cycle students. A statistically significant intestinal helmithic infection difference was observed between first and second cycle students (OR 3.70 and 95% CI is 0.69 – 5.60) (P = 0.205) (P = 0.001) (Table 2). All the other risk factors such as parents’ occupation, family size, finger nail status, shoe wearing habit, presence or absence of latrine, and water source for drinking were not associated with intestinal helminthiasis (P > 0.05).

**Table 2:** Univariate risk factor analysis for intestinal helminthic infection among students of Babile town, Eastern Ethiopia, 2012

Variables	Result of stool examination		OR(95% CI)	P-value
	Negative for Helminths	Positive for Helminths		
**Sex**				
Male	323(88.7%)	41(11.3%)	0.61(0.39, 0.96)	0.033
Female	232(82.9%)	48(17.1%)	1	
	**Negative for Helminths**	**Positive for Helminths**		
**Age range**				
5 - 14	508 (85.7%)	85 (14.3%)	1.97 (0.69, 5.60)	0.205
15 - 25	47 (92.2%)	4 (7.8%)	1	
	**Negative for Helminths**	**Positive for Helminths**		
**Grouped grade**				
First cycle	365 (82.4%)	78 (17.6%)	3.70 (1.92, 7.12)	0.001
Second cycle	190 (94.5%)	11 (5.5%)	1	

After adjusting for all predictors of helminthic infection, females were two times more likely to develop intestinal helminthic infection than males (Adjusted OR 1.67 and 95% C.I. is 1.04 – 2.67) (P = 0.033). Similarly, students of lower grade were four times more likely to develop helminthic infection than students of higher grade (Adjusted OR 3.75 and 95% C.I. is 1.65 – 8.55) and (P = 0.002) (Table 3).

**Table 3:** Parameter estimates from multivariable logistic regression model predicting the probability of helminthic infection among students of Babile town, Eastern Ethiopia, 2012

Predictors of helminthic infection	B	P-Value	Adjusted OR	95.0% C.I.	
				Lower	Upper
Sex (Male)			1.00		
Sex	0.51	0.033*	1.67	1.04	2.67
Age (Years)	-0.02	0.710	0.98	0.88	1.09
Grade of students (Second cycle)			1.00		
Grade of students	1.32	0.002*	3.75	1.65	8.55
Finger nail status (Trimmed)			1.00		
Finger nail status	-0.59	0.354	0.56	0.16	1.93
Latrine usage (Always)			1.00		
Latrine usage	0.50	.668	1.64	0.17	15.86
Hand washing after defecation (Yes)			1.00		
Hand washing after defecation	-0.51	0.666	0.60	0.06	6.02
Eating uncooked vegetables (No)			1.00		
Eating uncooked vegetables	0.26	0.707	1.30	0.33	5.07
Water for drinking (Tap water)			1.00		
Water for drinking	0.19	0.574	1.21	0.627	2.32

* P-value significant at <0.05

## Discussion

Helminths are the most common infectious agents of humans in developing countries and produce a global burden of disease that exceeds better-known conditions, including malaria and tuberculosis [25]. The inhabitants of thousands of rural, impoverished villages throughout the tropics and subtropics are often chronically infected with several different species of parasitic worms [26, 27]. For reasons not well understood, compared with any other age group, school-aged children (including adolescents) and preschool children tend to harbor the greatest numbers of intestinal worms [28]. Despite the fact mentioned above the prevalence rate of intestinal helminthiasis in present study was 13.8% the majority being mono-infection. This was happened due to the regular de-worming of schoolchildren that had already been started by the local Health Bureau (Personal Communication). The concerned health professionals of the Bureau is undertaking the de-worming campaign regularly every six month using a single day dose of 400mg Albendazole during the school days in collaboration with teachers, unit leaders and Directors of the two schools. It may also be due to the improved health service coverage by the local Health Buearu [23].

In the present study an overall prevalence of 13.8% intestinal helminthic infection was found. This finding was lower when compared with previous study conducted in the same area 11 years ago 27.2% [22]. This difference could be explained by the increased involvement of College of Health and Medical Sciences, Haramaya University through its normal teaching learning processes i.e. students assigned for Team Training Program (TTP) for the last many years might have contributed to the improvement of the health status of the community in the study area through time in addition to the de-worming campaign introduced in the area. It is also in disagreement with previous studies in Ethiopia; 26.9% among school children of community school at University of Gondar [17], and 47.1% among school children of Jimma zone [19]. The possible explanation for this difference could be better climatic and geographic condition of Babile town than Jimma and Gondar areas that prohibits the transmission of intestinal helminths.

In this study the prevalence of soil transmitted helminths was 0.47%; which was very much lower in comparison to different previous studies conducted in Ethiopia such as 14.2% in Babile [22], 38.4% in Jimma zone [19], 43.3% in Zarima, Gondar [18], 63% in Chencha, Southern Ethiopia (Ashenafi), and 66.5% in Delgi, Gondar [15]. This low prevalence could be due to the increased health service coverage by the local health Buearu in addition to the launching of periodic de-worming program by the Ethiopian government.

The predominant parasite detected in this study was H. nana with a rate of 13% which is almost similar to the study conducted among school children of Community school at the University of Gondar 13.8% [17]. However, it was slightly higher than the study conducted in the same area some 11 years ago with a rate of 10.1% [22]. This is may be associated with the life cycle of the parasite; it is one the auto-infecting helminths that can be transmitted by autoinfection means if and only if remained untreated. This finding revealed that despite periodic de-worming the prevalence of *H. nana* is on increase. This is because the de-worming focuses primarily on soil transmitted helminths by the known anthelminthic Albendazole which has little effect on eliminating *H. nana* on a single day dose administration. This increment in prevalence rate of *H. nana* shows absence of concern towards hymenolepiasis by the responsible bodies. This is an indication for the need to do further investigation on hymenolepiasis with its determinant factors for a plausible solution to be forwarded.

The observed 13% prevalence of Hymenolepiasis is in contrast with previous reports from different regions of Ethiopia; 0.8% in Chencha, Southern Ethiopia [16], 2.1% in Jimma zone [19], and 6.8% in Delgi, Gondar [15]. This difference may be explained by variations in socio-economic status, climatic and geographic condition of the study area as well as local endemicity of the study area for this particular parasite. In our study univariate analyses indicated that more female children 48 (17.1%) were infected with intestinal helminths than male children 41(11.3%) and found to be statistically significant (P = 0.033) (Table 2). This is in agreement with data obtained from a study in Nigeria; reported as female children (56.6%) were more infected than male children (46.4%) and the difference was statistically significant (P = 0.0019) [21]. However, it is in disagreement with Gondar's study reported that no statistical significant association was observed between males and females (p = 0.301) [17]. Higher helminthic infections were observed among the age range 5 – 14 compared to the age range 15 – 25 in the present study although the difference was not statistically significant (P = 0.205) (Table 2).

In the present study, a strong association between intestinal helminths and grades of students was detected (P = 0.018). That is, the lesser the grades of the students the more will be the intestinal parasites seen. This study also revealed that the existence of an overall intestinal helminthic infection difference between first and second cycle students (P = 0.001). This is in accordance with a previous report in Gondar town indicating that Children in grade one to grade three had a higher prevalence of intestinal helminthic infections than those in grades four to eight (p = 0.031) [17].

Of all the predictors of intestinal helminthic infection, sex is significantly associated with intestinal helminthic infection. Because logistic regression analysis showed that females are two times more likely to develop intestinal helminthic infection than males (Table-3). This could be due to carelessness and unhygienic habits practiced by female children than male children in Babile town.

Similarly, of all the predictors of intestinal helminthic infection, grade of the students is significantly associated with overall intestinal helminthic infection. Because logistic regression analysis showed that students of lower grade are four times more likely to develop intestinal helminthic infection than students of higher grade. This could be speculated by increase in awareness; as grade increases awareness increases so that students of higher grade will have less exposure to intestinal helminthic infection than students of lower grade (Table 3). Because students of higher grade are the more mature age group with a higher level of personal hygiene in contrast to students of lower grade.

## Conclusion

The current study identifies low prevalence of intestinal helminthiasis. Nearly one out of ten students was infected with intestinal helminths; almost all with *Hymenolepis nana*. Extremely low number of students almost one out of two hundred was infected with soil transmitted helminths. Female children are two times more likely to develop intestinal helminthic infection than male children. Students of lower grade are four times more likely to develop intestinal helminthic infection than students of higher grade. Generally health information dissemination is suggested to be given to students on how to protect themselves from intestinal helminthic infections with special emphasis for female children. It is also suggested that the local Education Bureau as well as the local Health Bureau need to provide safe learning environment especially for students of lower grade such as school sanitation. Since infection by *Hymenolepis nana* is a long term health problem in the area, provision of regular treatment to students of the town by anthelminthic drug of choice against hymenolepiasis is also recommended.
